# Mapping functions for the PHQ-9 and GAD-7 to generate EQ-5D-3L for economic evaluation

**DOI:** 10.1007/s10198-024-01692-0

**Published:** 2024-04-25

**Authors:** Clara Mukuria, Matthew Franklin, Sebastian Hinde

**Affiliations:** 1 Division of Population Health, Sheffield Centre of Health and Related Research, University of Sheffield, Sheffield, S1 4DA UK; 2 Centre for Health Economics, University of York, York, YO10 5DD UK

**Keywords:** Mapping, EQ-5D-3L, PHQ-9, GAD-7, Utilities, Economic evaluation

## Abstract

**Purpose:**

Generic preferenced-based measures, such as EQ-5D-3L, that are used to estimate quality adjusted life years (QALYs) for economic evaluation are not always available in clinical trials. Predicting EQ-5D-3L values from the commonly used Patient Health Questionnaire 9 (PHQ-9) and Generalised Anxiety Disorder-7 (GAD-7) would allow estimation of QALYs from such trials. The aim was to provide mapping functions to estimate EQ-5D-3L from PHQ-9 and GAD-7 to facilitate economic evaluation.

**Methods:**

Data was drawn from four trials of patients with symptoms of depression testing collaborative care or computerised cognitive behavioural therapy. Patients completed PHQ-9, GAD-7, and EQ-5D-3L at different timepoints. Mapping was undertaken using adjusted limited dependent variable mixture models (ALDVMM), ordinary least squares (OLS), and Tobit models based on PHQ-9, GAD-7 scores or questions, and age to predict EQ-5D-3L utilities. Models were selected based on mean error (ME), mean absolute error (MAE), root mean squared error (RMSE), model goodness of fit, and visual inspection of the predictions.

**Results:**

There were 5583 and 3942 observations for EQ-5D-3L combined with PHQ-9 and GAD-7 respectively. ALDVMM models had low ME ( ≤|0.0018|) and MAE ranging from 0.189 to 0.192, while RMSE was from 0.251 to 0.254 and had better predictions than OLS and Tobit models. ALDVMM models with four components based on PHQ-9 and GAD-7 scores are recommended for estimating EQ-5D-3L utilities.

**Conclusions:**

Recommended mapping functions provide users with an approach to estimate EQ-5D-3L utilities for economic evaluation using PHQ-9, GAD-7, or both scores where they have been used together.

**Supplementary Information:**

The online version contains supplementary material available at 10.1007/s10198-024-01692-0.

## Background

Many international health technology assessment organisations, such as the National Institute of Health and Care Excellence (NICE) in England [1], use economic evaluation, the comparative assessment of the costs and benefits of alternative interventions, to support resource allocation decisions in healthcare. The need for consistency and comparability in their recommendation decisions has resulted in the increased use of quality adjusted life years (QALYs) which combine length of life with health-related quality of life (HRQoL), measured using utilities, into a single metric. Utilities are generated using generic preference-based measures, which are applicable to any disease area, in contrast to condition-specific measures that have limited generalisability, and therefore risk commissioning decisions across disease areas being inconsistent [2]. The EQ-5D-3L [3] is one of the most widely used and preferred generic preference-based measures [4] and is currently NICE’s recommended measure for economic evaluation [1].

Generic preference-based measures are not always included in trials as condition-specific measures may be considered more informative. Reducing the number of measures also minimises the additional burden on patients. In mental health, there are a number of condition-specific HRQoL measures for common conditions such as depression and anxiety e.g., the Patient Health Questionnaire-9 (PHQ-9) [5] and the Generalised Anxiety Disorder-7 (GAD-7) [6]. These measures assess a patient’s mental health but are not designed to inform QALY estimation. Mapping between the condition-specific measure and a generic preference-based measure using regression analysis is one method for indirectly obtaining utilities. The mapping regression results can then be applied to other trials and settings where the preference-based measures are missing. NICE recommend that EQ-5D can be estimated from another measure using statistical mapping when EQ-5D is appropriate but not available in the relevant study [1].

A recent review [7] found only a limited number of published mapping studies using mental health measures, including the PHQ-9 and GAD-7 [8]. Most of the mapping studies in the review, including those looking at mental health measures, used ordinary least squares (OLS) regression as the regression approach [7]. There are limitations with using OLS as utilities are bounded, errors are not normally distributed, and measures such as EQ-5D-3L have trimodal distributions [9]. Alternative and more flexible approaches have been developed to address these concerns [10]. A more recent study employed equipercentile linking analysis to map from PHQ-9 to EQ-5D-3L [11] but the study was criticised for not following the most recent guidelines on mapping [12]. A different study provided mapping from the PHQ-9 and the GAD-7 to the EQ-5D-5L United States (US) utilities and the mapped EQ-5D-3L UK utilities using more appropriate mapping approaches [13]. EQ-5D-5L is a newer version of the EQ-5D [14] but the three-level version has been used in older trials and observational data where data may be drawn from to inform models. The EQ-5D-3L also continues to be recommended for use by NICE [1]. The utilities generated from the two EQ-5D versions are not equivalent therefore where analysts want to generate utilities from the PHQ-9 and the GAD-7 that are comparable to the EQ-5D-3L, an appropriate mapping algorithm is required. The objective of this study was therefore to address this gap in the literature by generating mapping functions between two commonly used measures of mental health, the PHQ-9 and GAD-7, and the EQ-5D-3L.

## Methods

### Data

The data was drawn from four trials: the Collaborative Care for Screen Positive Elders (CASPER) trial [15], CASPER PLUS [16], the Randomised Evaluation of the Effectiveness and Acceptability of Computerised Therapy (REEACT) trial [17] and REEACT 2 [18] (Supplementary Table S1). The CASPER trials were testing collaborative care whereas REEACT trials compared different computerised cognitive behavioural therapy (cCBT), with telephone facilitation in REEACT 2. The primary outcome measure across all trials was the PHQ-9 at 4 months. All trials included the EQ-5D-3L and three of the trials included the GAD-7 (Supplementary Table S1). CASPER and CASPER PLUS focused on older adults (aged 65 and over) while the REEACT trials were open to adults (aged 18 and over). Given the differences in the participants recruited into the trials, data was combined across the trials and three time points that were common across the trials (baseline, six and twelve months) to ensure variability. All participants gave informed consent for their anonymous data to be used in other research. NHS ethics was obtained for all the trials (see Supplementary Table 1).

**Table 1:** Summary statistics, stratified into those completing PHQ-9 and EQ-5D-3L or GAD-7 and EQ-5D-3L

Completed PHQ-9					Completed GAD-7				
	Mean	SD	Min	Max		Mean	SD	Min	Max
*Total*									
EQ-5D-3L	0.613	0.29	− 0.594	1	EQ-5D-3L	0.59	0.29	− 0.594	1
PHQ-9	10.3	6.35	0	27	GAD-7	7.44	5.67	0	21
PHQ-9 severity (n %)					GAD-7 severity (n %)				
None	1246	22.3			None	1429	36.3		
Mild	1478	26.5			Mild	1222	31.0		
Moderate	1397	25.0			Moderate	724	18.4		
Severe	1462	26.2			Severe	567	14.4		
*Baseline*					*Baseline*				
Age	57.89	20.66	18	98	Age	65.58	18.29	18	98
Female (n, %)	1443	62.8			Female (n, %)	969	60.9		
White (n, %)	2243	97.9			White (n, %)	1555	98		

### Measures

#### EQ-5D-3L

The EQ-5D-3L questionnaire has five dimensions: mobility, self-care, usual activities, pain, and anxiety/depression. Patients are asked to report their level of problems (no problems, some/moderate problems, or severe/extreme problems) with their responses describing the patient’s health state. Utilities for each state have been elicited using time trade-off (TTO) in the UK based on representative sample (n = 2,997) of non-institutionalised adults [19]. These UK values range from − 0.594 to 1 with a score of zero considered equivalent to death and 1 perfect health.

#### PHQ 9 and GAD-7

The PHQ-9 is the nine-item depression module of the Patient Health Questionnaire [5] covering aspects related to interest/pleasure, depression/hopelessness, trouble with sleep, tiredness/lack of energy, appetite loss/overeating, feelings of failure, trouble concentrating, restlessness/fidgeting, and suicidal/self-harm thoughts. Items are completed on a 4-point scale from 0 to 3 (“not at all”, “several days”, “more than half the days”, and “nearly every day”). The total scores range from 0 to 27 with higher scores indicating depression. A score of 10 or greater has been validated to assess depression diagnosis while cut points of 5, 10, and 15 were used to represent mild, moderate, and severe levels of depression [5].

The GAD-7 items cover core symptoms of generalized anxiety disorder which include feeling nervous/anxious, unable to stop worrying, worrying too much, trouble relaxing, restless, annoyed/irritable, and “afraid something awful will happen” [6]. Response options are the same as the PHQ-9. Total scores range from 0 to 21 with higher scores indicating anxiety. As with PHQ-9 a score of 10 or more has been validated to assess cases of GAD while cut points of 5, 10, and 15 were used to represent mild, moderate and severe levels of GAD [6].

PHQ-9 and GAD-7 are frequently used in the same population and a combination of both measures—the Patient Health Questionnaire Anxiety and Depression Scale (PHQ-ADS)—has been found to be a valid and reliable measure of depression and anxiety [20, 21].

### Analysis

#### Preliminary assessment

As recommended by the ISPOR Guide for Mapping [9], assessment of the distribution of all the measures at overall score and item/dimension level was undertaken to inform the analysis including which regression methods could be used. The relationship between EQ-5D-3L and the PHQ-9 and GAD-7 was assessed using Spearman’s rank correlation for ordinal variables (dimensions/items) and Pearson’s correlation for continuous variables (utilities/total scores). Correlations were judged based on recommended cut-offs of 0.1 to 0.29 (small), 0.3 to 0.49 (medium) and 0.5 or above (large) [22]. Mean EQ-5D-3L utilities were also plotted grouped by either PHQ-9 or GAD-7 scores.

#### Mapping analysis

Mapping can be undertaken to predict the utilities or the dimensions of the measure e.g. mobility, self-care etc. The predictors can be the total scores (from either PHQ-9 or GAD-7) or the item responses. For the total scores, squared, or cubic terms may also be included to address non-linearity. Item responses can be treated as either continuous or dummy variables. As there are only 4 severity levels in the PHQ-9 and GAD-7 questions, using dummy variables would be more appropriate but this depends on the distribution across severity levels. Age and gender have also been recommended as additional variables as they are commonly included in other datasets where mapping algorithms are applied [9].

As both PHQ-9 and GAD-7 are frequently used in the same population, we maximised our available data by modelling using both measures together and then each measure independently. The models that could be estimated included:

1. Total scores of both PHQ-9 and GAD-7
2. Total scores of both PHQ-9 and GAD-7 with squared terms
3. PHQ-9 and GAD-7 items (dummy variables)
4. PHQ-9 and GAD-7 items (continuous variables
5. PHQ-9 total scores
6. PHQ-9 total scores with square terms
7. PHQ-9 items (dummy variables)
8. PHQ-9 items (continuous variables)
9. GAD-7 total scores
10. GAD-7 total scores with square terms
11. GAD-7 items (dummy variables)
12. GAD-7 items (continuous variables)

Age was included in every model.

Utilities are continuous values but they are bounded within the range defined by the value set e.g., -0.594 to 1 for the EQ-5D-3L UK values with other characteristics such as a large proportion at a value of 1, a skewed distribution and multi-modal distributions within the data [10]. Alternative models have been used to address the specific nature of utilities e.g., Tobit to address the bounded range or two-part models to address distribution issues [7]. However, many of these techniques only address one aspect. The adjusted limited dependent variable mixture model (ALDVMM) was developed to address all the issues related to utility mapping including the bounded range, the mix of distributions and skewed distribution which are particularly an issue for EQ-5D-3L UK values [10]. The ALDVMM estimates utilities based on a mixture of normal distributions (referred to as components), the number of which are specified by the user, which are then adjusted based on the upper and lower bounds of the values being estimated. Any gaps in the utilities can also be included e.g., between the highest value and next feasible value and, separately, whether to explicitly predict the probability of component membership [10]. ALDVMM models use maximum likelihood in the estimation; it is possible to identify local solutions which are not global solutions, different search options were used to mitigate against this problem. Only models using the total scores (1, 5 and 9) were fitted for ALDVMM models as the inclusion of additional components increased the degrees of freedom required to fit models and therefore increased the sample size required to estimate models with confidence. Two to four components were tested alongside estimation of probability of component membership using the same variables used to estimate utilities. Squared terms for PHQ-9 and GAD-7 were not statistically significant in linear models and there was a risk of overfitting in the ALDVMM models therefore they were not included in these models.

Linear models remain the most popular approaches to undertaking mapping [7] therefore an Ordinary Least Squares (OLS) and Tobit regression, which allow the upper and lower bounds of the utility score to be taken into account, were estimated for comparison purposes. All 12 model specifications were fitted for OLS and Tobit. Given that ALDVMM addresses all the problems in a single model that other methods address separately, no other methods were tested. An alternative approach, response mapping, involves predicting the probability of being in different levels of each dimension of the EQ-5D-3L [23]. It therefore requires distribution across the three levels of the EQ-5D-3L in each of the five dimensions. However, in the trials used for this study, there were very few respondents (n < 30) at the lowest levels for mobility and self-care (see Supplementary Table S2), therefore response mapping was not undertaken.

#### Selecting mapping functions

The aim of mapping is to predict utilities therefore one approach to assess models is on how well they predict utilities. Mean error (ME), the mean absolute error (MAE) and the Root Mean Squared Error (RMSE), which rely on the difference between predicted and observed utilities, were used to assess the models, with smaller errors preferred. Mean predicted values and the predicted range was also assessed. The distribution of the predicted values was also assessed against the observed data using a cumulative distribution plot and based on groups of the PHQ-9 or GAD-7 total scores [24]. Goodness of fit of the models was assessed using the Akaike information criteria (AIC), which measures the information lost in a model and the Bayesian information criteria (BIC), which adjusts for the number of parameters. Lower values of AIC and BIC are preferred. All core variables (e.g., total scores and items) were retained regardless of statistical significance as recommended in the ISPOR Guide for Mapping [9]. Only those respondents who had complete cases were included in the analysis to allow comparison across different regression models.

All analysis was undertaken using Stata MP 17.0.

## Results

### Preliminary assessment

**Fig. 1:**
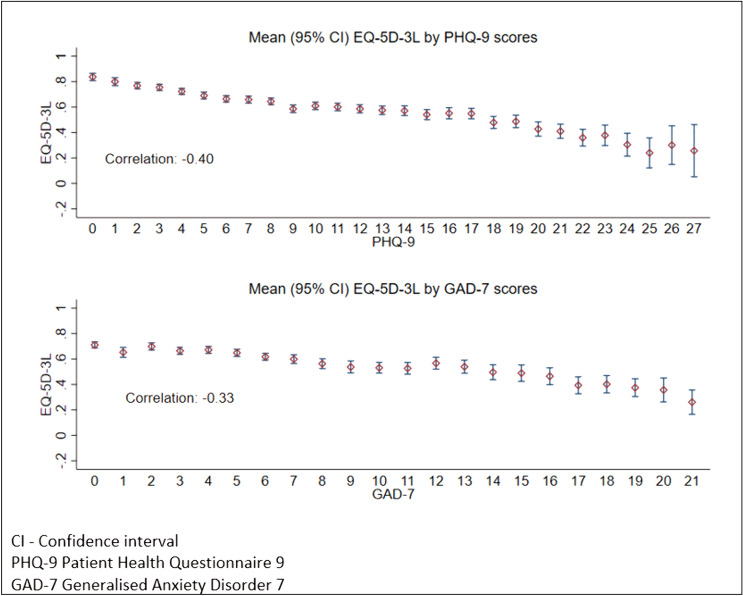
Mean EQ-5D-3L utilities by PHQ-9 and GAD-7 scores

There were 5583 and 3942 observations for EQ-5D-3L and PHQ-9 or GAD-7 respectively and 3902 observations with EQ-5D-3L, PHQ-9 and GAD-7. Descriptive summaries of the data are reported in Table 1. EQ-5D-3L exhibited a mixture of distributions (Supplementary Figure S1) but there were few participants at the extremes (1 participant at −0.594, 4.3 and 3.8% at 1 for the PHQ-9 and GAD-7 samples respectively (Supplementary Table S2)). There was a medium strength relationship (0.3 < *rho* < 0.5) between PHQ-9, GAD-7, and EQ-5D-3L utilities with lower values but wider variation as PHQ-9/GAD-7 scores increased (Fig. 1). PHQ-9 and GAD-7 questions had medium to large correlations with EQ-5D-3L utilities and the Depression or Anxiety dimension (Supplementary Table S3). There were small to medium correlations with usual activities while there were small to little or no relationship (*rho* ≤ 0.1) with mobility, self-care and pain/discomfort dimensions (Supplementary Table S3).

### Mapping

**Fig. 2:**
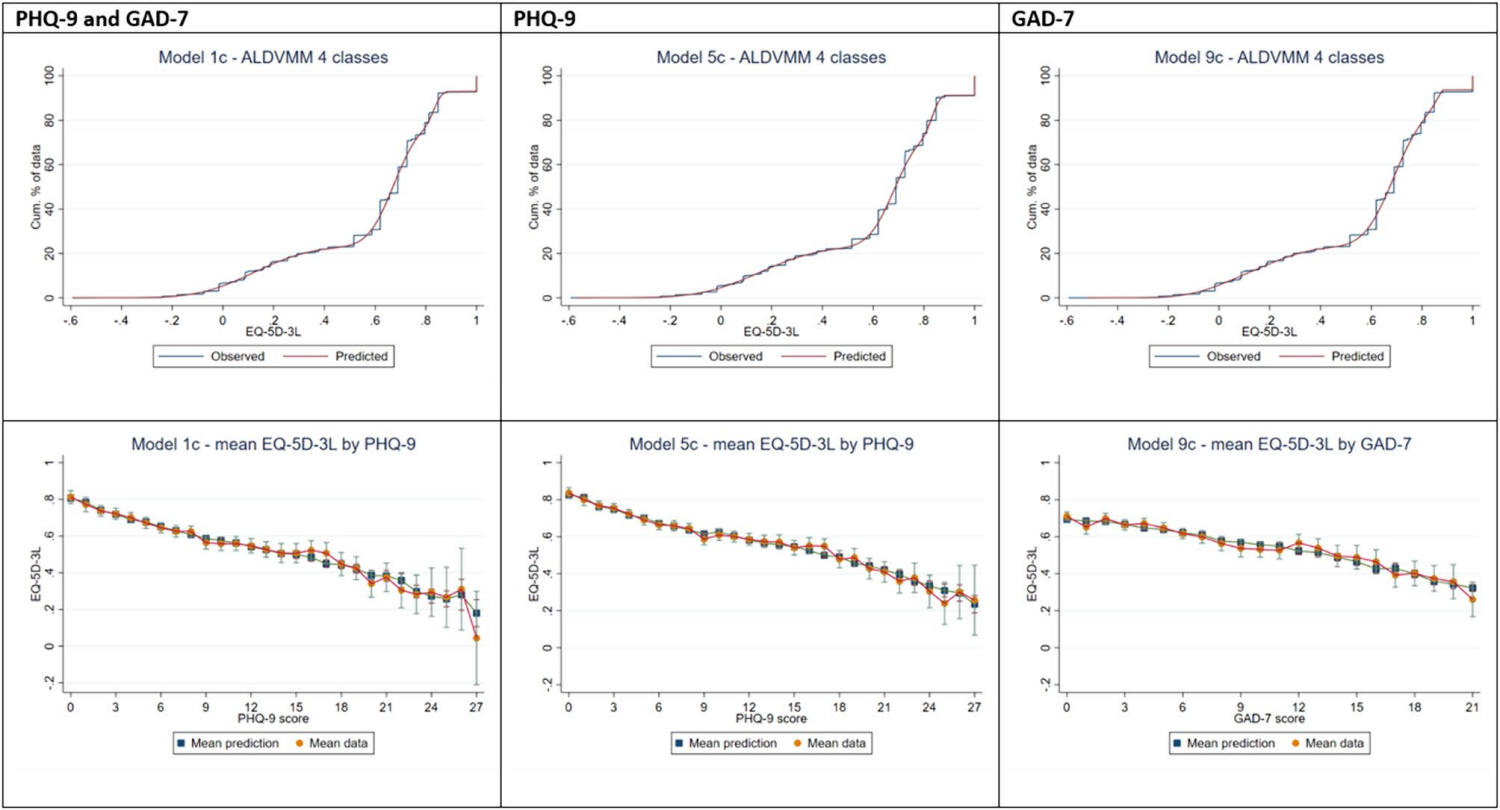
Best fitting ALDVMM models

Different ALDVMM models based on PHQ-9 and GAD-7 scores combined (1a to 1l, Supplementary Table S4) had low ME ( ≤|0.0018|and MAE ranging from 0.189 to 0.192 while RMSE was from 0.251 to 0.254. The mean EQ-5D-3L predictions ranged from 0.591 to 0.593 which was comparable to the observed mean of 0.591 but none of the models predicted the full range with predictions ranging from 0.041 to 0.963 compared to observed values of -0.594 to 1. The best fitting model based on AIC and BIC was model 1c which had the same main and probability predictors (PHQ-9, GAD-7, and age) and four components. Figure 2 shows the cumulative distribution of the observed and predicted EQ-5D-3L utilities from the best fitting ALDVMM model showing that predictions based on model 1c tracked closely with the observed values. Predicted mean EQ-5D-3L based on the PHQ-9 scores were similar to the observed values for less severe PHQ-9 scores with more variation as PHQ-9 scores increased (Fig. 2).

ALDVMM models fitted using PHQ-9 (5a to 5f) or GAD-7 (9a to 9f) scores had low ME ( ≤|0.0016|) (Supplementary Table S4). The mean EQ-5D-3L predictions ranged from 0.612–0.616 for the PHQ-9 models (observed: 0.613) and 0.589–0.592 for the GAD-7 models (observed: 0.590). The best fitting models based on AIC and BIC were models 5c and 9c for PHQ-9 and GAD-7 respectively and Fig. 2 shows that the cumulative distribution and mean EQ-5D-3L predictions indicating good predictions.

For comparison and illustration, OLS and Tobit models were also fitted (models 1–12) with different predictors (Supplementary Table S5). Although OLS models predict the mean EQ-5D-3L accurately, they predicted values over 1 for most of the models with the exception of GAD-7 models 10 and 11. On the other hand, Tobit models had the largest ME (0.0034–0.0036) across all the models and this was reflected in the predicted mean EQ-5D-5L mean which were not as accurate as the ALDVMM or OLS models (Supplementary Table S5). The OLS and Tobit models that used the individual questions from the PHQ-9 and GAD-7 as dummy variables tended to have smaller MAE and RMSE but these models had poor predictions based on the cumulative distributions of predicted EQ-5D-3L (see Supplementary Figures S3 and S4) although mean predictions by PHQ-9 and GAD-7 scores were reasonable.

### Selected model

Based on the predictions and their distribution, the best fitting ALDVMM models are recommended. Users can choose to use both PHQ-9 and GAD-7 scores where they have both been used in the same population or either one. We provide a look-up table (Online Supplement) to allow users to identify EQ-5D-3L utilities based on these PHQ-9, GAD-7 and age. For example, based on scores of 15 for both PHQ-9 and GAD-7, a 40-year-old would have a utility value of 0.587 but if their scores were 10 for both measures, then their utility score would be 0.696—which would represent a gain of 0.109 (Table 2). If only one of the measures was available e.g. PHQ-9, then the predicted utilities would be 0.609 and 0.705 (gain 0.096) respectively for scores of 15 and 10.

**Table 2:** Example EQ-5D-3L predictions at ages 25, 40, and 65

Age	PHQ-9 score	GAD-7 score	EQ-5D-3L (1)		EQ-5D-3L (2)		EQ-5D-3L (3)	
				Difference		Difference		Difference
25	15	15	0.675		0.683		0.632	
	10	10	0.756	0.081	0.765	0.082	0.752	0.121
40	15	15	0.587		0.609		0.565	
	10	10	0.696	0.109	0.705	0.096	0.693	0.127
65	15	15	0.430		0.478		0.429	
	10	10	0.570	0.140	0.595	0.116	0.561	0.132

EQ-5D-3L (1)—prediction from both PHQ-9 and GAD-7 scores

EQ-5D-3L (1)—prediction from PHQ-9 scores only

EQ-5D-3L (3)—prediction from GAD-7 scores only

## Discussion

This study aimed to estimate mapping function between the PHQ-9 and GAD-7 using patient data. ALDVMM models were estimated as they take into account the specific nature of EQ-5D-3L data and are therefore recommended for mapping [24]. These models performed well in terms of ME and replicating the distribution of the EQ-5D-3L data especially compared to other simpler approaches such as OLS and Tobit. The best fitting ALDVMM models were selected to allow estimation of utilities from both or either PHQ-9 and GAD-7 along with age.

However, none of the models could estimate the full range observed in the data. Across the ALDVMM models the smallest MAE and RMSE were 0.189 and 0.250 which still represents a large difference between observed and predicted values. There may be a number of explanations for this. Although EQ-5D-3L utilities had the typical bimodal distribution, there was only one participant at the lowest value and the mass at 1 was not as large as that observed in other studies which can be over 20% of the sample. There were few observations that had values below 0, with most observations lying between 0.5 and 0.9. This meant that there were less observations to fit the model in the lower values of the EQ-5D-3L. There were medium strength correlations between EQ-5D-3L and the PHQ-9 and GAD-7 total scores, but most of the overlap was driven by the overlap between the EQ-5D-3L anxiety/depression dimension and the condition-specific questions. Furthermore, although those with higher severity in either the PHQ-9 or the GAD-7 had lower EQ-5D-3L utilities, there was high variability within each severity level. Mapping cannot account for this lack of overlap.

In the most recently published work, equipercentile linking was used to map EQ-5D-3L utilities to PHQ-9 scores, but it did not account for the variability in EQ-5D-3L utilities that are observed at different values of the PHQ-9 [11]. For example, at baseline, individuals with a PHQ-9 score of 1 to 5 were assigned an EQ-5D-3L score of 1. Furthermore, follow-up scores were assigned lower EQ-5D-3L utilities for those scoring a PHQ-9 score that was higher than 2 e.g. those with a score of 3 to 5 were assigned a score of 0.91, 0.89 and 0.88 respectively. This implies that an individual who had the same PHQ-9 score from baseline to follow-up would have experienced a drop in utility if their PHQ-9 score was greater than 2. Furukawa et al. [11] explained these drops in utility on the basis of dissatisfaction in remaining at the same symptomatic point—although scores in this lower range would not be considered strong markers of symptoms. In the current study, we combine baseline and follow-up data in the analysis to avoid potentially problematic assumptions regarding how to assign individuals at follow-up.

There is evidence that EQ-5D-3L is a valid measure for assessing depression and to some extent anxiety [8] but its correlation with other mental health measures was small to medium (− 0.2 to − 0.49) [25]. The newer version of the EQ-5D, the EQ-5D-5L [14], was developed with an increased number of severity levels from 3 to 5 to address concerns with sensitivity especially at the mild end. EQ-5D-5L may therefore be better suited for assessing patients with anxiety and depression than EQ-5D-3L. However, medium strength correlations (− 0.39 to − 0.41) have also been reported between EQ-5D-5L and PHQ-9 and GAD-7 in patients who have anxiety and depression which may indicate that increasing the number of severity levels does not necessarily increase convergent validity [26]. Mapping algorithms have been developed to the EQ-5D-5L based on the US value set as well as when scored based on mapping to the EQ-5D-3L UK value set [13]. This current study has the advantage of mapping directly to the EQ-5D-3L UK values.

### Strengths and limitations

The mapping was undertaken using methods that were designed to address the specific nature of EQ-5D data and selection of models was based on the latest mapping guidance [9, 24]. Trial data from different trials was combined, increasing the sample size and the population in which the mapping algorithms are fitted. The CASPER trials [15, 16] included just older people but combining these with the REEACT [17, 18] trials ensures that potential users are not restricted from using the mapping algorithms due to differences in ages of the populations where they want to apply the results.

However, EQ-5D-3L utilities were estimated rather than the newer EQ-5D-5L. EQ-5D-3L has been used in previous trials and continues to be the recommended utilities in England and Wales [1] therefore the mapping results are useful in this context. Despite these strengths, there were limitations associated with mapping as there are always differences between predicted and observed values. Furthermore, although it is useful to map using specific measures that are used in trials such as the PHQ-9 and the GAD-7, the narrow focus of these measures means that other aspects of broader HRQoL may not be fully reflected in the estimated utilities [2]. It is therefore always preferable to collect data directly from patients where possible.

## Conclusion

Recommended mapping methods have been used to generate results that allow the estimation of EQ-5D-3L utilities from PHQ-9 and GAD-7 scores where EQ-5D-3L is not available. Users can predict utilities from either or both the clinical measures using the look up table that we have provided.

## Supplementary Information

Below is the link to the electronic supplementary material.

Supplementary file 1 (DOCX 727 KB)

Supplementary file 2 (XLSX 1728 KB)

## Data Availability

Data were drawn from clinical trials at the author institution and are therefore not freely available.
