# Supplementary material for: Mapping functions for the PHQ-9 and GAD-7 to generate EQ-5D-3L for economic evaluation
Source: Eur J Health Econ. 2024 Apr 25;26(1):63–70. doi: 10.1007/s10198-024-01692-0 (PMC11743390; doi:10.1007/s10198-024-01692-0)
Supplement: Supplementary file 1 — (DOCX 727 KB) [file 10198_2024_1692_MOESM1_ESM.docx]

**Electronic supplementary material 1**

**Mapping functions for the PHQ-9 and GAD-7 to generate EQ-5D-3L for economic evaluation**

Supplementary Table S1: Data sources

| **Trial** | **CASPER** | **CASPER PLUS** | **REEACT** | **REACT 2** |
| --- | --- | --- | --- | --- |
| **Design** | Pragmatic, multicentre, two-arm, parallel RCT | Pragmatic, multicentre, two-arm, parallel RCT | Pragmatic, multicentre three arm, parallel RCT |  |
| **Sample** | Older adults (65+) with lower severity depressive symptoms | Older adults (65+) with major depressive symptoms | Adults (18+) with symptoms of depression | Adults (18+) with symptoms of depression |
| **Inclusion** | Depressive symptoms based on DSM-IV criteria using the Mini International Neuropsychiatric Interview (vs 5.0) | | PHQ-9 score ≥10 and who had access to the internet | PHQ-9 score ≥10 and <3 for question 9 (suicidal thoughts) |
| **Exclusions** | Patients with alcohol dependency, psychosis, recent suicidal risk, significant cognitive impairment, recent bereavement, or terminal illness on clinical grounds. Patients receiving psychological therapy | | Patients who were actively suicidal; experiencing psychotic symptoms; depressed in the postnatal period; or had recently been bereaved. | Patients who were actively suicidal; diagnosed with psychotic depression; had a primary diagnosis of alcohol or drug abuse, had given birth within the last year; or had recently been bereaved |
| **Interventions** | Collaborative care vs usual care from GP | Collaborative care vs usual care from GP | cCBT1 (Beating the Blues) or cCBT2 (MoodGYM) or usual care | cCBT with telephone vs cCBT |
| **Measures** | PHQ-9, GAD-7 and EQ-5D-3L | PHQ-9, GAD-7 and EQ-5D-3L | PHQ-9 and EQ-5D-3L | PHQ-9, GAD-7 and EQ-5D-3L |
| **Time points** | Baseline, 4 and 12 months | Baseline, 4, 12 and 18 months | Baseline, 4, 12 and 24 months | Baseline, 4 and 12 months |
| **Sample size** | 705 | 485 | 691 | 369 |
| **Randomisation** | collaborative care = 344; usual care = 361 | collaborative care = 249; usual care = 236 | cCBT1 = 210; cCBT2= 242; usual care = 239 | cCBT with telephone = 187; cCBT = 182 |
| **Randomisation dates** | May 2011-July 2013 | September 2012 – August 2014 | August 2009 – March 2011 | June 2011 – April 2013 |
| **NHS Ethics** | NHS Leeds East Research Ethics Committee (REC) reference no. 10/H1306/61 | NHS Leeds East REC reference no. 10/H1306/61 | NHS Leeds East REC | Bradford REC reference no. 10/H1302/95 |

CASPER - Collaborative Care for Screen Positive Elders; REEACT - Randomised Evaluation of the Effectiveness and Acceptability of Computerised Therapy; cCBT – computerised Cognitive Behavioural Therapy; DSM-IV - Diagnostic and Statistical Manual of Mental Disorders (Fourth Edition)

Supplementary Table S2: Distribution at item level for EQ-5D-3L, PHQ-9 and GAD-7

|  | PHQ-9 sample | | | |  |  | GAD-7 sample | | | |
| --- | --- | --- | --- | --- | --- | --- | --- | --- | --- | --- |
|  | 0 | 1 | 2 | 3 |  |  | 0 | 1 | 2 | 3 |
|  |  |  |  |  |  |  |  |  |  |  |
| Mobility |  | 3,176 | 2,394 | 13 |  |  |  | 1,875 | 2,056 | 11 |
| Self-care |  | 4,748 | 802 | 33 |  |  |  | 3,245 | 668 | 29 |
| Usual activities |  | 2,349 | 2,912 | 322 |  |  |  | 1,518 | 2,180 | 244 |
| Pain/ discomfort |  | 1,688 | 3,302 | 593 |  |  |  | 995 | 2,436 | 511 |
| Anxiety/ depression |  | 1,638 | 3,326 | 619 |  |  |  | 1,212 | 2,363 | 367 |
|  |  |  |  |  |  |  |  |  |  |  |
| PHQ9_01 | 1,386 | 2,238 | 1,203 | 756 |  | GAD7_01 | 1,081 | 1,675 | 677 | 509 |
| PHQ9_02 | 1,301 | 2,365 | 1,141 | 776 |  | GAD7_02 | 1,195 | 1,486 | 651 | 610 |
| PHQ9_03 | 1,001 | 1,603 | 1,264 | 1,715 |  | GAD7_03 | 996 | 1,575 | 700 | 671 |
| PHQ9_04 | 505 | 2,016 | 1,417 | 1,645 |  | GAD7_04 | 1,192 | 1,438 | 671 | 641 |
| PHQ9_05 | 2,100 | 1,508 | 1,044 | 931 |  | GAD7_05 | 2,081 | 1,094 | 469 | 298 |
| PHQ9_06 | 2,046 | 1,700 | 1,020 | 817 |  | GAD7_06 | 1,236 | 1,546 | 675 | 485 |
| PHQ9_07 | 2,143 | 1,702 | 1,021 | 717 |  | GAD7_07 | 1,992 | 1,089 | 480 | 381 |
| PHQ9_08 | 3,237 | 1,240 | 718 | 388 |  |  |  |  |  |  |
| PHQ9_09 | 4,126 | 985 | 331 | 141 |  |  |  |  |  |  |

PHQ-9 items:

1. interest or pleasure,
2. depression/hopelessness,
3. trouble with sleep,
4. tiredness/lack of energy,
5. appetite loss/overeating,
6. feelings of failure,
7. trouble concentrating,
8. restlessness/inactivity (moving/speaking so slowly others noticed)
9. suicidal/self-harm thoughts

GAD-7:

1. feeling nervous/anxious,
2. unable to stop worrying,
3. worrying too much,
4. trouble relaxing,
5. restless,
6. annoyed/irritable,
7. afraid something awful would happen

Supplementary Table S3: Correlation at item and total score/utility level for EQ-5D-3L, PHQ-9 and GAD-7

|  | EQ-5D-3L | Mobility | Self-care | Usual activities | Pain/ discomfort | Anxiety/ depression |
| --- | --- | --- | --- | --- | --- | --- |
|  |  |  |  |  |  |  |
| phq9_01 | -0.3226* | 0.0480* | 0.1112* | 0.2828* | 0.0834* | 0.4962* |
| phq9_02 | -0.3103* | -0.0272* | 0.0811* | 0.2290* | 0.0446* | 0.5956* |
| phq9_03 | -0.2505* | 0.0025 | 0.0614* | 0.1826* | 0.1171* | 0.3617* |
| phq9_04 | -0.3415* | 0.1012* | 0.1334* | 0.3057* | 0.1759* | 0.3950* |
| phq9_05 | -0.2447* | -0.0005 | 0.0756* | 0.2158* | 0.0935* | 0.3612* |
| phq9_06 | -0.2183* | -0.0978* | 0.0432* | 0.1658* | -0.0131 | 0.5256* |
| phq9_07 | -0.2620* | -0.0280* | 0.1099* | 0.2196* | 0.0560* | 0.4411* |
| phq9_08 | -0.3037* | 0.0764* | 0.1552* | 0.2430* | 0.1296* | 0.3618* |
| phq9_09 | -0.2138* | 0.0093 | 0.0932* | 0.1540* | 0.0415* | 0.3617* |
|  |  |  |  |  |  |  |
|  |  |  |  |  |  |  |
| gad7_01 | -0.2568* | -0.0880* | 0.0765* | 0.1506* | 0.0157 | 0.6015* |
| gad7_02 | -0.2901* | -0.0536* | 0.0725* | 0.1718* | 0.0494* | 0.6047* |
| gad7_03 | -0.2825* | -0.0541* | 0.0566* | 0.1612* | 0.0624* | 0.5922* |
| gad7_04 | -0.2710* | -0.0478* | 0.0781* | 0.1647* | 0.0825* | 0.5208* |
| gad7_05 | -0.2349* | -0.0149 | 0.0787* | 0.1231* | 0.0947* | 0.4031* |
| gad7_06 | -0.2081* | -0.1016* | 0.0461* | 0.1192* | 0.0459* | 0.4724* |
| gad7_07 | -0.2585* | -0.0335* | 0.0624* | 0.1598* | 0.0472* | 0.5066* |

0.1 to <0.3 (small), 0.3 to <0.5 (medium) and ≥ 0.5 (large)

* Statistically significant at 5% level

PHQ-9 items:

1. interest or pleasure,
2. depression/hopelessness,
3. trouble with sleep,
4. tiredness/lack of energy,
5. appetite loss/overeating,
6. feelings of failure,
7. trouble concentrating,
8. restlessness/inactivity (moving/speaking so slowly others noticed)
9. suicidal/self-harm thoughts

GAD-7:

1. feeling nervous/anxious,
2. unable to stop worrying,
3. worrying too much,
4. trouble relaxing,
5. restless,
6. annoyed/irritable,
7. afraid something awful would happen

Supplementary Table S4: Fit statistics and estimated EQ-5D-3L utilities (ALDVMM)

|  |  |  |  |  |  |  |  |  |  |  | Predicted EQ-5D-3L | | |
| --- | --- | --- | --- | --- | --- | --- | --- | --- | --- | --- | --- | --- | --- |
| Model | Main predictors | Probability predictors | Components | log likelihood | df | AIC | BIC | ME | MAE | RMSE | Mean | Min | Max |
| ALDVMM models - PHQ-9 and GAD7 | |  |  |  |  |  |  |  |  |  |  |  |  |
| 1a | phq9 gad7 age | phq9 gad7 age | 2 | 755.7 | 14 | -1483 | -1396 | 0.0003 | 0.189 | 0.252 | 0.591 | 0.031 | 0.936 |
| 1b | phq9 gad7 age | phq9 gad7 age | 3 | 884.5 | 23 | -1723 | -1579 | 0.0008 | 0.189 | 0.252 | 0.590 | 0.042 | 0.934 |
| 1c | phq9 gad7 age | phq9 gad7 age | 4 | 993.8 | 32 | -1924 | -1723 | 0.0001 | 0.191 | 0.251 | 0.591 | 0.056 | 0.963 |
| 1d | phq9 gad7 age | phq9 age | 2 | 736.3 | 13 | -1447 | -1365 | 0.0004 | 0.191 | 0.253 | 0.591 | 0.043 | 0.934 |
| 1e | phq9 gad7 age | phq9 age | 3 | 864.2 | 21 | -1686 | -1555 | 0.0009 | 0.191 | 0.253 | 0.590 | 0.056 | 0.932 |
| 1f | phq9 gad7 age | phq9 age | 4 | 941.3 | 29 | -1825 | -1643 | 0.0010 | 0.191 | 0.253 | 0.590 | 0.071 | 0.932 |
| 1g | phq9 gad7 age | phq9 gad7 | 2 | 714.6 | 13 | -1403 | -1322 | -0.0009 | 0.190 | 0.253 | 0.592 | 0.089 | 0.915 |
| 1h | phq9 gad7 age | phq9 gad7 | 3 | 814.4 | 21 | -1587 | -1455 | -0.0018 | 0.190 | 0.254 | 0.593 | 0.090 | 0.911 |
| 1i | phq9 gad7 age | phq9 gad7 | 4 | 857.9 | 29 | -1658 | -1476 | -0.0007 | 0.191 | 0.253 | 0.592 | 0.014 | 0.922 |
| 1j | phq9 gad7 age | phq9 | 2 | 706.4 | 12 | -1389 | -1314 | -0.0007 | 0.191 | 0.254 | 0.592 | 0.094 | 0.914 |
| 1k | phq9 gad7 age | phq9 | 3 | 803.2 | 19 | -1568 | -1449 | -0.0017 | 0.191 | 0.255 | 0.593 | 0.096 | 0.911 |
| 1l | phq9 gad7 age | phq9 | 4 | 858.4 | 26 | -1665 | -1502 | 0.0004 | 0.192 | 0.256 | 0.591 | 0.142 | 0.901 |
| ALDVMM models - PHQ-9 | |  |  |  |  |  |  |  |  |  |  |  |  |
| 5a | phq9 age | phq9 age | 2 | 937.5 | 11 | -1853 | -1780 | 0.0000 | 0.188 | 0.250 | 0.613 | 0.055 | 0.920 |
| 5b | phq9 age | phq9 age | 3 | 1120.9 | 18 | -2206 | -2086 | 0.0012 | 0.189 | 0.250 | 0.612 | 0.052 | 0.916 |
| 5c | phq9 age | phq9 age | 4 | 1275.4 | 25 | -2501 | -2335 | 0.0002 | 0.189 | 0.250 | 0.613 | 0.080 | 0.955 |
| 5d | phq9 age | phq9 | 2 | 884.3 | 10 | -1749 | -1682 | -0.0016 | 0.189 | 0.252 | 0.615 | 0.121 | 0.905 |
| 5e | phq9 age | phq9 | 3 | 975.2 | 16 | -1918 | -1812 | -0.0024 | 0.190 | 0.251 | 0.615 | 0.156 | 0.926 |
| 5f | phq9 age | phq9 | 4 | 1158.9 | 22 | -2274 | -2128 | -0.0032 | 0.191 | 0.252 | 0.616 | 0.230 | 0.928 |
| ALDVMM models - GAD-7 | |  |  |  |  |  |  |  |  |  |  |  |  |
| 9a | gad7 age | gad7 age | 2 | 601.95 | 11 | -1182 | -1113 | 0.0004 | 0.198 | 0.261 | 0.590 | 0.124 | 0.909 |
| 9b | gad7 age | gad7 age | 3 | 692.00 | 18 | -1348 | -1235 | 0.0004 | 0.198 | 0.261 | 0.590 | 0.137 | 0.912 |
| 9c | gad7 age | gad7 age | 4 | 811.57 | 25 | -1573 | -1416 | 0.0008 | 0.198 | 0.261 | 0.589 | 0.149 | 0.909 |
| 9d | gad7 age | gad7 | 2 | 565.73 | 10 | -1111 | -1049 | -0.0007 | 0.199 | 0.263 | 0.591 | 0.215 | 0.877 |
| 9e | gad7 age | gad7 | 3 | 661.41 | 16 | -1291 | -1190 | -0.0016 | 0.199 | 0.263 | 0.592 | 0.221 | 0.873 |
| 9f | gad7 age | gad7 | 4 | 728.17 | 22 | -1412 | -1274 | -0.0003 | 0.199 | 0.263 | 0.590 | 0.166 | 0.841 |

Green highlights best estimate or fit statistic

df degrees of freedom; AIC Akaike Information Criteria; BIC Bayesian Information Criteria; ME mean error; MAE mean absolute error; RMSE Root Mean Squared Error; Min Minimum; Max Maximum

Supplementary Table S5: Fit statistics and estimated EQ-5D-3L values (Ordinary Least Squares), (OLS and Tobit)

|  |  |  |  |  |  |  |  |  |  | Predicted EQ-5D-3L | | |
| --- | --- | --- | --- | --- | --- | --- | --- | --- | --- | --- | --- | --- |
| Model | Main predictors | N | Log likelihood | DF | AIC | BIC | ME | MAE | RMSE | Mean | Min | Max |
| OLS models | |  |  |  |  |  |  |  |  |  |  |  |
| 1 | PHQ-9 GAD-7 age | 3902 | -154.24 | 4 | 316 | 342 | 0 | 0.192 | 0.252 | 0.591 | 0.071 | 1.067 |
| 2 | PHQ-9 GAD-7 squared terms age | 3902 | -149.67 | 6 | 311 | 349 | 0 | 0.191 | 0.251 | 0.591 | 0.046 | 1.054 |
| 3 | PHQ-9 and GAD-7 dummy items age | 3902 | -84.10 | 50 | 268 | 582 | 0 | 0.187 | 0.247 | 0.591 | -0.002 | 1.060 |
| 4 | PHQ-9 and GAD-7 continuous items age | 3902 | -126.83 | 18 | 290 | 403 | 0 | 0.190 | 0.250 | 0.591 | 0.051 | 1.049 |
| 5 | PHQ-9 age | 5583 | -180.67 | 3 | 367 | 387 | 0 | 0.190 | 0.250 | 0.613 | 0.126 | 1.025 |
| 6 | PHQ-9 squared terms age | 5583 | -179.73 | 4 | 367 | 394 | 0 | 0.190 | 0.250 | 0.613 | 0.105 | 1.015 |
| 7 | PHQ-9 dummy items age | 5583 | -119.69 | 29 | 297 | 490 | 0 | 0.187 | 0.247 | 0.613 | 0.078 | 1.021 |
| 8 | PHQ-9 continuous items age | 5583 | -148.41 | 11 | 319 | 392 | 0 | 0.189 | 0.248 | 0.613 | 0.104 | 1.010 |
| 9 | GAD-7 age | 3942 | -303.10 | 3 | 612 | 631 | 0 | 0.199 | 0.261 | 0.590 | 0.181 | 1.004 |
| 10 | GAD-7 squared terms age | 3942 | -299.48 | 4 | 607 | 632 | 0 | 0.198 | 0.261 | 0.590 | 0.149 | 0.988 |
| 11 | GAD-7 dummy items age | 3942 | -287.61 | 23 | 621 | 766 | 0 | 0.198 | 0.260 | 0.590 | 0.142 | 0.992 |
| 12 | GAD-7 continuous items age | 3942 | -301.51 | 9 | 621 | 678 | 0 | 0.199 | 0.261 | 0.590 | 0.181 | 1.005 |
| Tobit models | |  |  |  |  |  |  |  |  |  |  |  |
| 1 | PHQ-9 GAD-7 age | 3902 | -626.21 | 5 | 1262 | 1294 | 0.0034 | 0.191 | 0.251 | 0.588 | 0.029 | 0.946 |
| 2 | PHQ-9 GAD-7 squared terms age | 3902 | -622.71 | 7 | 1259 | 1303 | 0.0034 | 0.191 | 0.251 | 0.588 | 0.050 | 0.948 |
| 3 | PHQ-9 and GAD-7 dummy items age | 3902 | -562.01 | 51 | 1226 | 1546 | 0.0033 | 0.187 | 0.247 | 0.588 | -0.017 | 0.954 |
| 4 | PHQ-9 and GAD-7 continuous items age | 3902 | -601.76 | 19 | 1242 | 1361 | 0.0033 | 0.190 | 0.250 | 0.588 | 0.022 | 0.943 |
| 5 | PHQ-9 age | 5583 | -990.53 | 4 | 1989 | 2016 | 0.0035 | 0.190 | 0.250 | 0.609 | 0.076 | 0.934 |
| 6 | PHQ-9 squared terms age | 5583 | -989.11 | 5 | 1988 | 2021 | 0.0036 | 0.191 | 0.250 | 0.609 | 0.103 | 0.939 |
| 7 | PHQ-9 dummy items age | 5583 | -935.25 | 30 | 1931 | 2129 | 0.0035 | 0.188 | 0.247 | 0.610 | 0.055 | 0.945 |
| 8 | PHQ-9 continuous items age | 5583 | -964.98 | 12 | 1954 | 2033 | 0.0035 | 0.189 | 0.248 | 0.610 | 0.067 | 0.931 |
| 9 | GAD-7 age | 3942 | -785.72 | 4 | 1579 | 1605 | 0.0035 | 0.199 | 0.261 | 0.587 | 0.150 | 0.917 |
| 10 | GAD-7 squared terms age | 3942 | -784.91 | 5 | 1580 | 1611 | 0.0035 | 0.199 | 0.261 | 0.587 | 0.136 | 0.913 |
| 11 | GAD-7 dummy items age | 3942 | -773.74 | 24 | 1595 | 1746 | 0.0035 | 0.198 | 0.260 | 0.587 | 0.123 | 0.915 |
| 12 | GAD-7 continuous items age | 3942 | -784.08 | 10 | 1588 | 1651 | 0.0035 | 0.200 | 0.261 | 0.586 | 0.155 | 0.918 |

df degrees of freedom; AIC Akaike Information Criteria; BIC Bayesian Information Criteria; ME mean error; MAE mean absolute error; RMSE Root Mean Squared Error; Min Minimum; Max Maximum

Supplementary Figure S1: Distribution of EQ-5D-3L utilities, PHQ-9 scores and GAD-7 scores

| 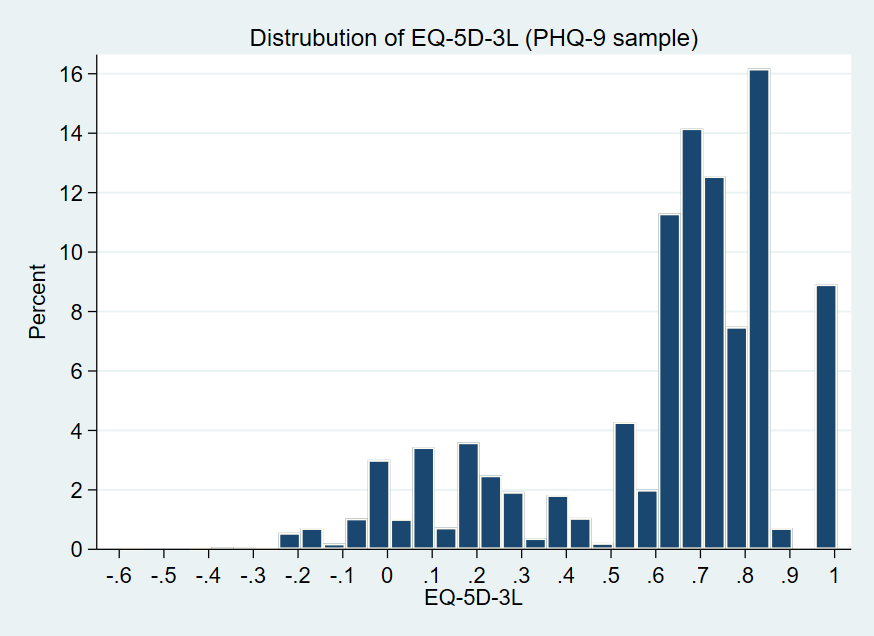 | 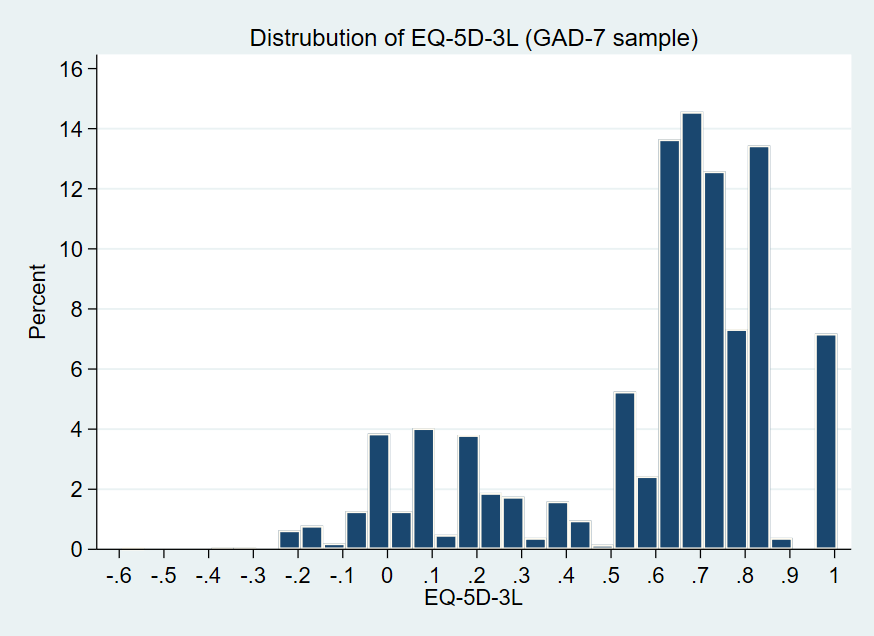 |
| --- | --- |
| 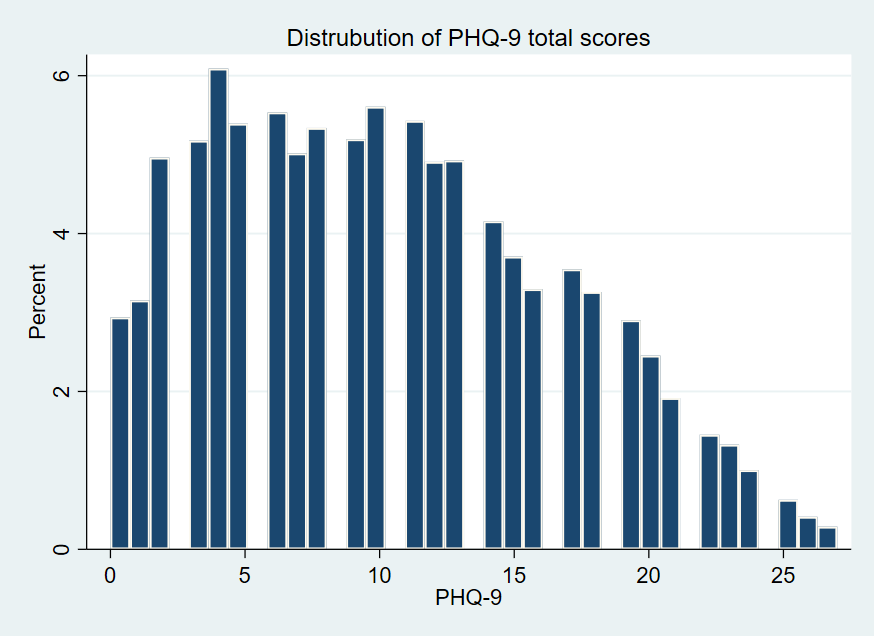 | 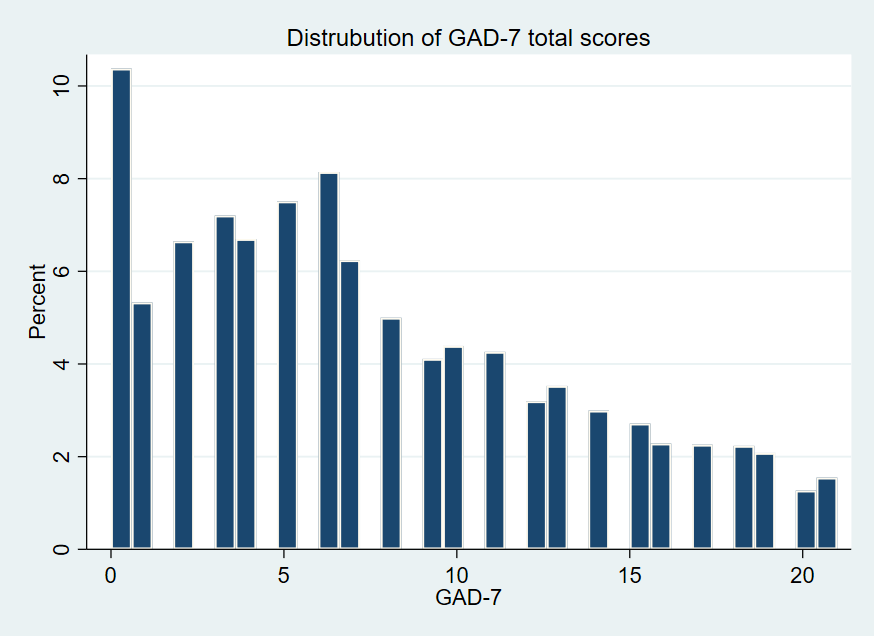 |

Supplementary Figure S2: EQ-5D-3L by PHQ-9 and GAD-7 severity

| 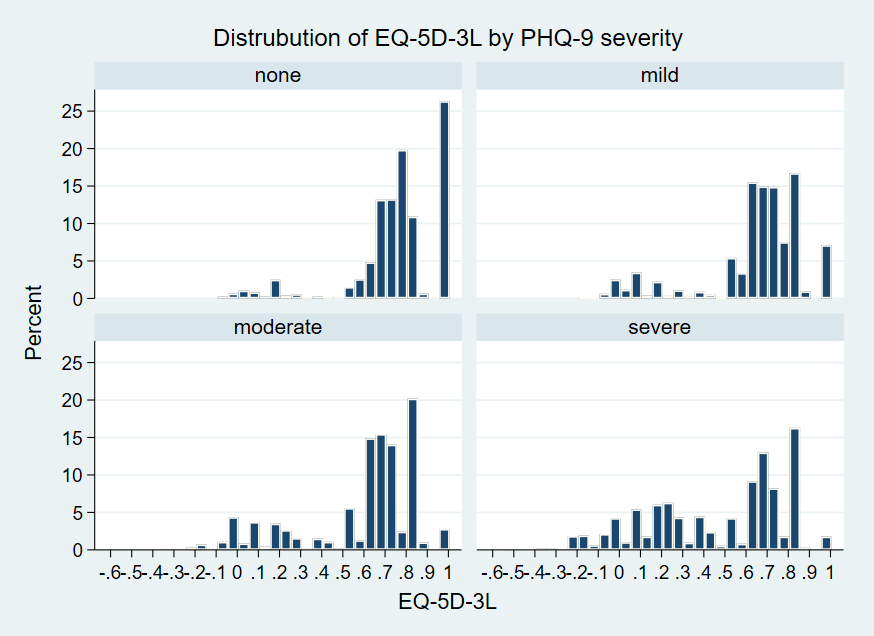 | 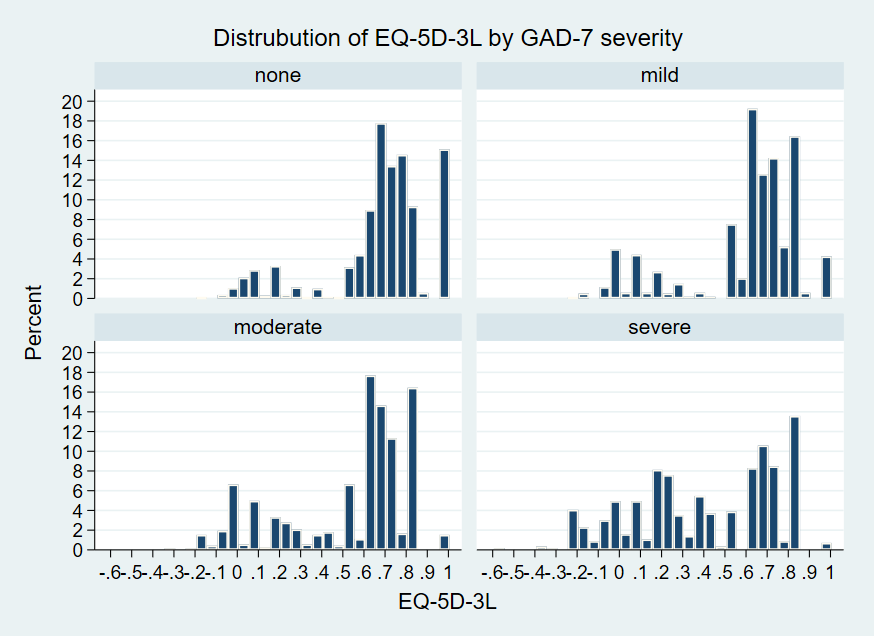 |
| --- | --- |

|  |  | EQ-5D-3L | |  |  |  |  |  | EQ-5D-3L | |
| --- | --- | --- | --- | --- | --- | --- | --- | --- | --- | --- |
| PHQ-9 | N | Mean | SD |  |  |  | GAD-7 | N | Mean | SD |
|  |  |  |  |  |  |  |  |  |  |  |
| None (0-4) | 1246 | 0.77 | 0.22 |  |  |  | None (0-4) | 1429 | 0.68 | 0.25 |
| Mild (5-9) | 1478 | 0.65 | 0.24 |  |  |  | Mild (5-9) | 1222 | 0.60 | 0.27 |
| Moderate (10-15) | 1397 | 0.59 | 0.28 |  |  |  | Moderate (10-15) | 724 | 0.53 | 0.29 |
| Severe (15-21) | 1462 | 0.47 | 0.32 |  |  |  | Severe (15-21) | 567 | 0.40 | 0.33l |

Supplementary Figure S3: Cumulative distribution and distribution by mean for observed and predicted EQ-5D-3L selected OLS models

| **PHQ-9 and GAD-7** | **PHQ-9** | **GAD-7** |
| --- | --- | --- |
| 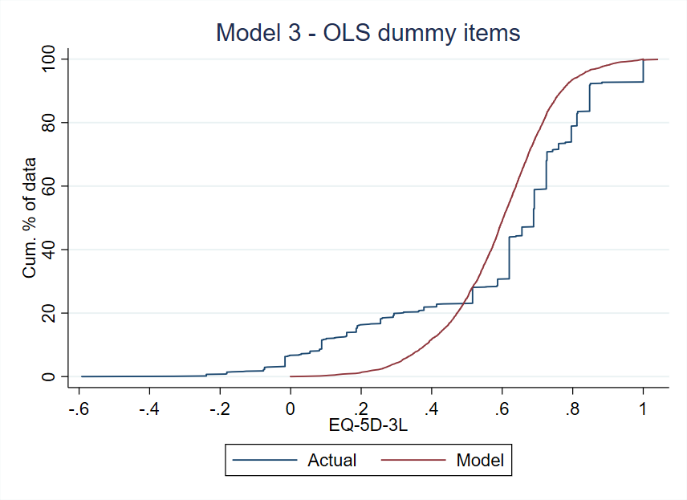 | 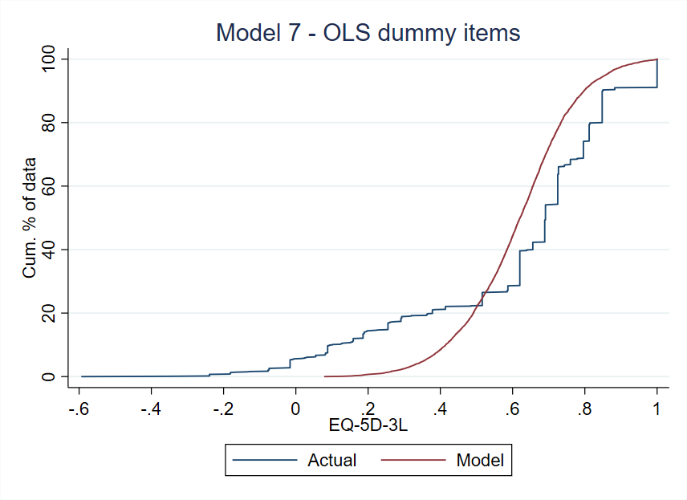 | 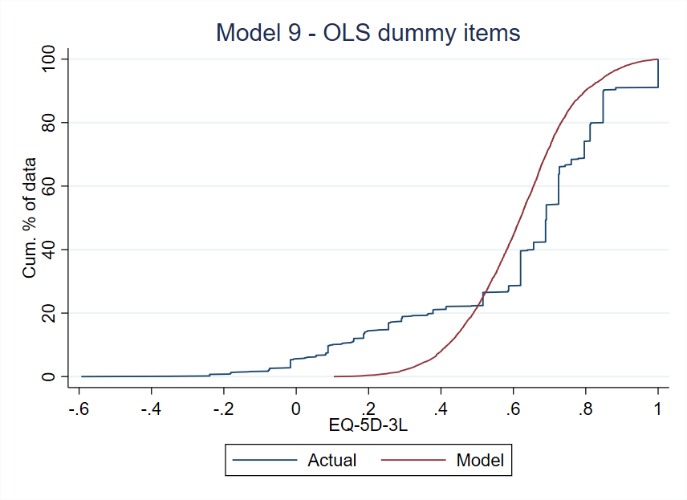 |
| 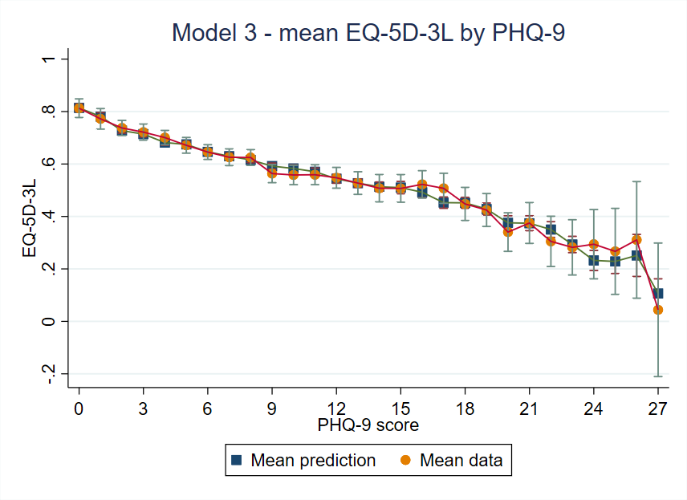 | 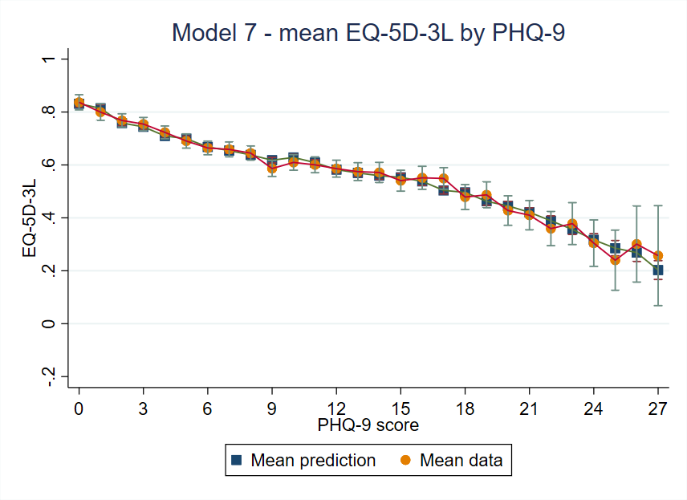 | 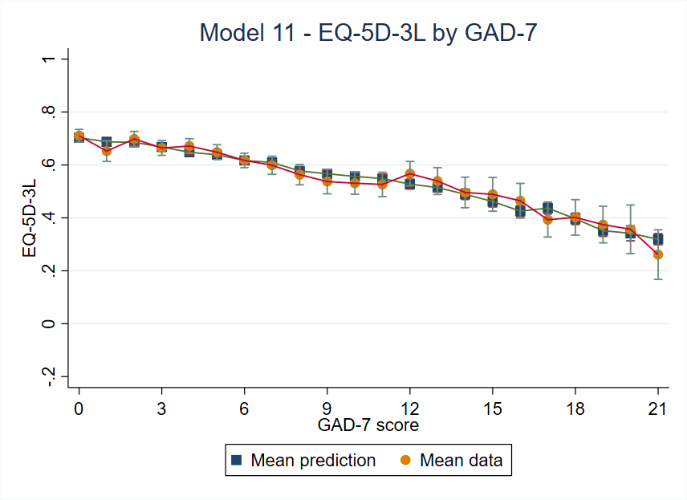 |

Supplementary Figure S4: Cumulative distribution and distribution by mean for observed and predicted EQ-5D-3L selected Tobit models

| **PHQ-9 and GAD-7** | **PHQ-9** | **GAD-7** |
| --- | --- | --- |
| 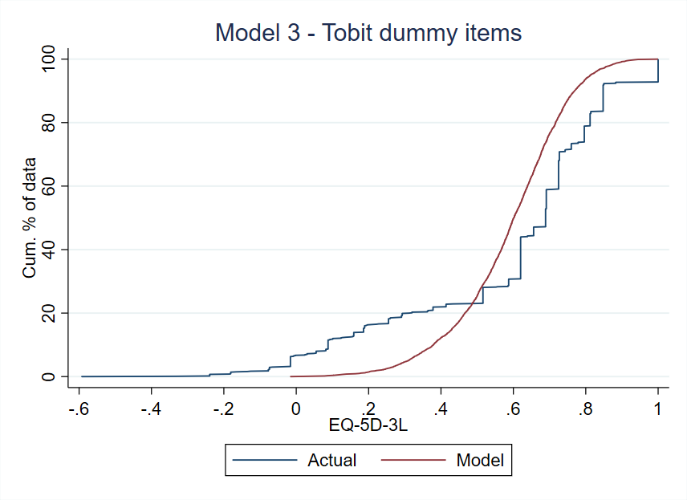 | 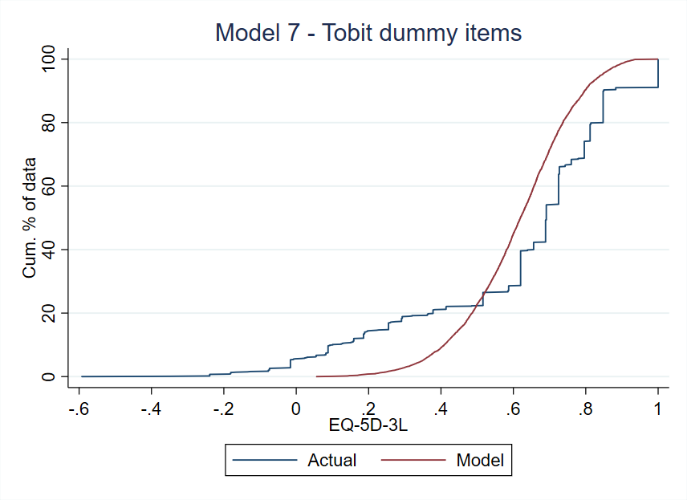 | 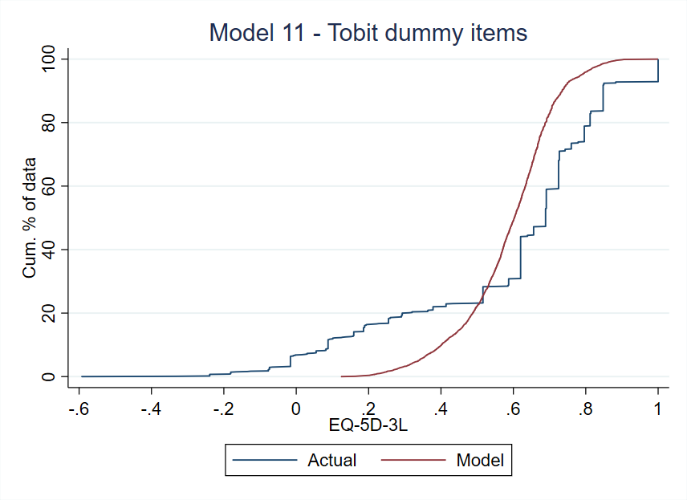 |
| 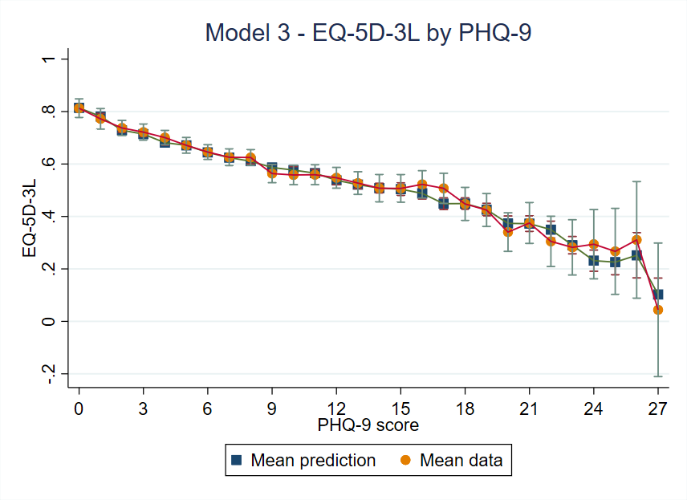 | 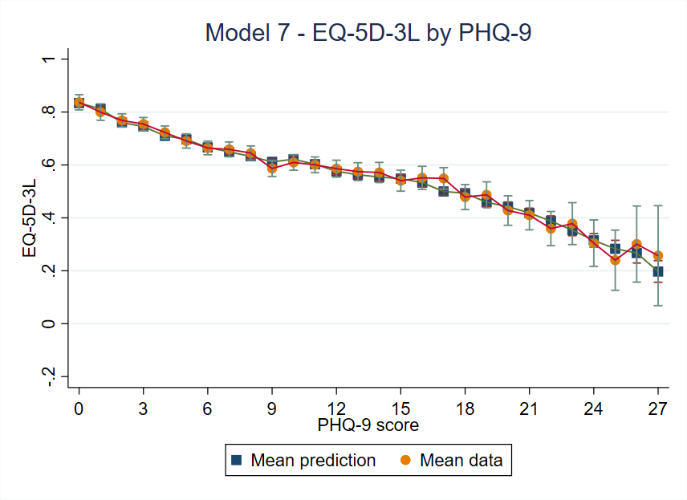 | 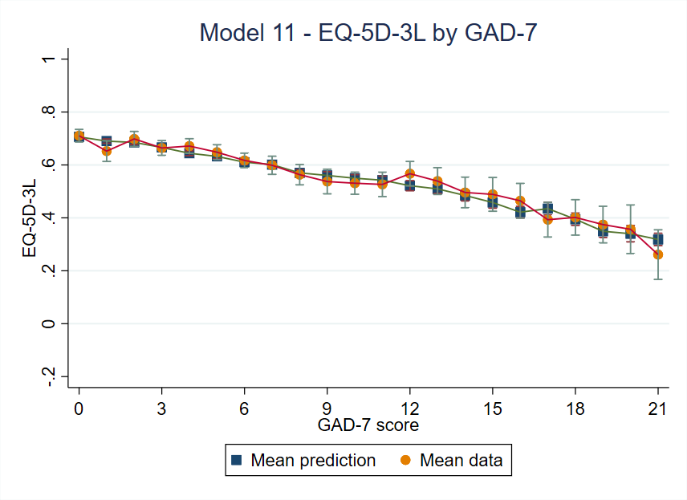 |
